# A Call for Standardized Interview Offer Dates in the Neurology Residency Application Process

**DOI:** 10.1212/NE9.0000000000200077

**Published:** 2023-06-02

**Authors:** Joseph R. Geraghty

**Affiliations:** From the Medical Scientist Training Program, University of Illinois College of Medicine, Chicago; Hospital of the University of Pennsylvania, Philadelphia.

The transition to virtual interviewing during the coronavirus disease 2019 pandemic has had desired and undesired effects on the residency application process. By eliminating the immense monetary cost, effort, and time away from rotations required to travel the country, virtual interviewing has helped applicants navigate the residency application process in a more equitable and affordable manner.^1,2^ There is also potential for new challenges, including the rising number of applications that may limit a more holistic review of individual applications, reliance on quality internet access, time-zone differences, and inability for applicants to visit a hospital or city in person.^1,2^

Another potential, unintended consequence of virtual interviews is the exacerbation of “interview hoarding,” a phenomenon whereby more competitive applicants hold on to a disproportionately large number of interview slots, often at the expense of fellow applicants. Concern for increased interview hoarding was large enough in 2020 for the Association of American Medical Colleges (AAMC) to issue a letter urging medical students to “consider releasing some interviews if you are holding more than needed.”^3^ Indeed, concerns of interview hoarding is one of several reasons that the Coalition of Physician Accountability's Undergraduate Medical Education-to-Graduate Medical Education Review Committee highlighted the need for development and implementation of standards in the interview offer and acceptance process.^4^ Several solutions have been proposed to address this problem, including interview caps, a lottery, or even a separate interview-match process.^5^ Each of these has benefits and challenges, but most would require substantial changes to the residency application system. One potential solution that could be readily implemented before the next application cycle is establishing standardized interview offer dates across all residency programs.

Currently, candidates are invited to interview in neurology and several other specialties on a first come, first serve, rolling manner. After submission of the residency application in late September, applicants can receive an interview offer on any day and at any time between early October and January. Applicants anxiously await interview offers and may need to check their phones or emails, often disrupting their clinical education.^6^ Applicants are incentivized to accept every initial interview offer they receive, regardless of their interest in each program, because there is no guarantee that they will receive offers later in the process from more highly desired programs. If initial interview offers are scheduled early enough, applicants may have already completed interviews at less desired programs before receiving offers at more desired programs, further contributing to interview hoarding.

With standardized interview offers on predetermined dates, neurology applicants would be empowered to make more informed decisions about which offers to accept and far less incentivized to hoard interviews. Promising data to support this claim already exist outside of neurology. For the 2019–2020 application cycle, the Association of Professors of Gynecology and Obstetrics (APGO) and the Council on Resident Education in Obstetrics and Gynecology (CREOG) recommended that programs adopt 2 standardized interview offer dates each 1 week apart (October 8 and 15, 2019).^7^ This recommendation was followed by approximately 50% of obstetrics and gynecology (OB/GYN) programs and resulted in a decrease in the number of applicants with excess interviews.^7,8^ Following the standardized interview offer dates, programs were allowed to extend rolling offers as needed. Programs also set a final deadline of October 1 for applicants to submit applications to ensure that all applications were submitted by the time of interview offers. Applicants were also required to respond to an offer within 72 hours, allowing time for programs to consider alternative applicants before the next offer date. These changes allowed for an improved distribution of interviews, reduced applicant anxiety, and added an element of predictability to the process.

Computer simulation models have also shown that interview hoarding contributes to both unmatched students and unfilled programs because programs had less ability to determine sincere interest and students outside of the top applicant pool were not offered interviews.^9^ Within OB/GYN, applicants receiving standardized interview dates had 4-fold less odds of completing ≥20 interviews.^8^ This is even more significant given that nearly 40% of MD applicants to OB/GYN received less than the recommended 12 interviews required for a high chance of matching. Similar initiatives have been discussed in other specialties^10^; however, to date, no such call for high-value interviewing processes has been discussed in the neurology literature. According to the AAMC Electronic Residency Application Service, the average number of applications received by neurology compared with OB/GYN programs in 2022 were comparable (659.5 vs 669.6, respectively), which may diminish concerns of meeting a similar deadline for interview offers.^11^ However, the effects of program size and the optimal time line for neurology applications and interviews require further consideration. Similar to the approach by APGO and CREOG, the American Academy of Neurology, the American Neurological Association, and/or the Association for University Professors of Neurology could adopt similar recommendations for programs to consider in upcoming cycles.

Further research on the benefits of standardized interview offer dates within neurology is necessary. Even more broadly, the AAMC and the National Resident Matching Program should provide more detailed data on the number of programs offering standardized interview offer dates across graduate medical education. The time has come for neurology to join this effort toward more standardized interview processes. Establishing uniform interview offer dates is a potential way to start.

## Appendix Author

**Table:**

Name	Location	Contribution
Joseph R. Geraghty, MD, PhD	Medical Scientist Training Program, University of Illinois College of Medicine, Chicago; current affiliation: Hospital of the University of Pennsylvania, Philadelphia	Drafting/revision of the manuscript for content, including medical writing for content; major role in the acquisition of data; study concept or design; analysis or interpretation of data
